# The Blood Component Iron Causes Neuronal Apoptosis Following Intracerebral Hemorrhage via the PERK Pathway

**DOI:** 10.3389/fneur.2020.588548

**Published:** 2020-12-07

**Authors:** Muyao Wu, Rong Gao, Baoqi Dang, Gang Chen

**Affiliations:** ^1^Department of Rehabilitation, Zhangjiagang TCM Hospital Affiliated to Nanjing University of Chinese Medicine, Suzhou, China; ^2^Department of Neurosurgery, Zhangjiagang TCM Hospital Affiliated to Nanjing University of Chinese Medicine, Suzhou, China; ^3^Department of Neurosurgery and Brain and Nerve Research Laboratory, The First Affiliated Hospital of Soochow University, Suzhou, China

**Keywords:** blood components, iron, intracerebral hemorrhage, PERK pathway, apoptosis

## Abstract

PERK signaling pathway plays an important role in neuronal apoptosis after Intracerebral hemorrhage (ICH). ICH can cause the release of blood components into the brain. However, which component in the blood plays a major role still unclear. This study was designed to investigate the activation of the PERK pathway in different blood components after ICH and explore which components have major relationships with neuronal apoptosis. Eighty-five Sprague–Dawley rats were used to establish an ICH model. Western blot (WB) and immunofluorescence (IF) were used to evaluate the expression of the PERK pathway. TUNEL staining, FJC staining and neurological score were used to evaluate neuronal apoptosis and necrosis after ICH. The results showed that protein levels of p-PERK and p-eIF2α were upregulated following ICH with the injection of Fe^3+^ and Fe^2+^ after 48 h. Then, deferoxamine (DFX) was used to study the roles of Fe^3+^ in ICH through the PERK signaling pathway. The results showed that injection of DFX reversed increasing protein levels and prevented neuronal apoptosis. Thus, iron plays an important role in ICH through the PERK signaling pathway. Furthermore, the reduction of iron demonstrates neuroprotective effects in ICH. This suggests that targeting intervention of the iron and PERK pathway could be an effective treatment strategy to improve ICH prognosis.

## Introduction

Intracerebral hemorrhage (ICH), a common subtype of hemorrhagic stroke, has high morbidity/mortality with an estimated yearly worldwide prevalence of 16/100,000 (1). Studies have shown that inflammation, oxidative stress, cytotoxicity and excitotoxicity caused by plasma components and red blood cell (RBC) lysates were the main factors causing secondary brain injury (SBI) following ICH, ultimately leading to brain edema and brain parenchymal damage (2, 3). ICH can cause disorders in cell metabolism, activating endoplasmic reticulum stress (ERs) and the unfolded protein response (UPR), eventually reestablish cellular homeostasis. The cell death program is activated when too much stress to restore homeostasis (4). ICH can cause the release of blood components into the brain, such as erythrocytes and fibrinogen. Erythrocytes start to lyse 1 day following ICH and continues over days to weeks (5). When erythrocytes break down, hemoglobin (Hb) is metabolized into heme, which is further degraded into biliverdin, ferrous iron, and CO by heme oxygenase-1 (HO-1) and heme oxygenase-2 (HO-2) (6, 7). Lysis of RBCs leads to an increase in iron content surrounding the hematoma. Iron can initiate the release of large amounts of reactive oxygen species (ROS), induce the production of lipid ROS. The excessive accumulation of lipid ROS causes intracellular oxidative stress response, which leads to the damage of proteins and lipids, and ultimately results in neuronal ferroptosis and cell death. Additionally, it can lead to secondary brain injuries such as brain edema, intracranial hematoma and irreversible neurological deficits following ICH (8–10). It has been indicated that RBC lysis, with the release of iron, play a significant role in the development of hydrocephalus after intraventricular hemorrhage (11). The cytotoxicity of iron generated by hemolysis is associated with oxidative stress and inflammatory response (12). Studies over the past decade had found that prevention of iron-mediated cytotoxicity is significant for the treatment of SBI after ICH. Chelation of iron with deferoxamine (DFX) reduced brain injury in animals (13, 14).

ERs caused by misfolded/unfolded accumulation of proteins is one of the main mechanisms leading to neuronal apoptosis. When ERs occurs, UPR can remove misfolded/unfolded proteins, allowing it to possibly play a significant role in cell survival (15, 16). The UPR is triggered by three types of ER stress sensor proteins: PERK, inositol requiring kinase 1 (IRE1) and activating transcription factor six (ATF6). PERK acts as a central pressure sensor in ER, restoring homeostasis by performing adaptive programs through protein translation and induction of the activating ATF4 (17). The inhibition of PERK signaling pathway can reduce SBI following ICH by suppressing apoptosis (18, 19). However, it is not clear which component of the hematoma plays the key role following ICH and whether it is related to the PERK pathway activated by the UPR.

Given its widespread prevalence and high mortality/morbidity, exploration of the pathogenesis and mechanisms of ICH, as well as identification of new biomarkers and therapeutic targets is of the utmost importance. This study investigated the roles of different blood components, especially iron, in SBI following ICH, via the PERK signaling pathway. Specifically, a combination of DFX and Fe^3+^ was used to investigate whether iron in the blood plays an important role in neuronal apoptosis following ICH.

## Materials and Methods

### Experimental Design

**Figure 1:**
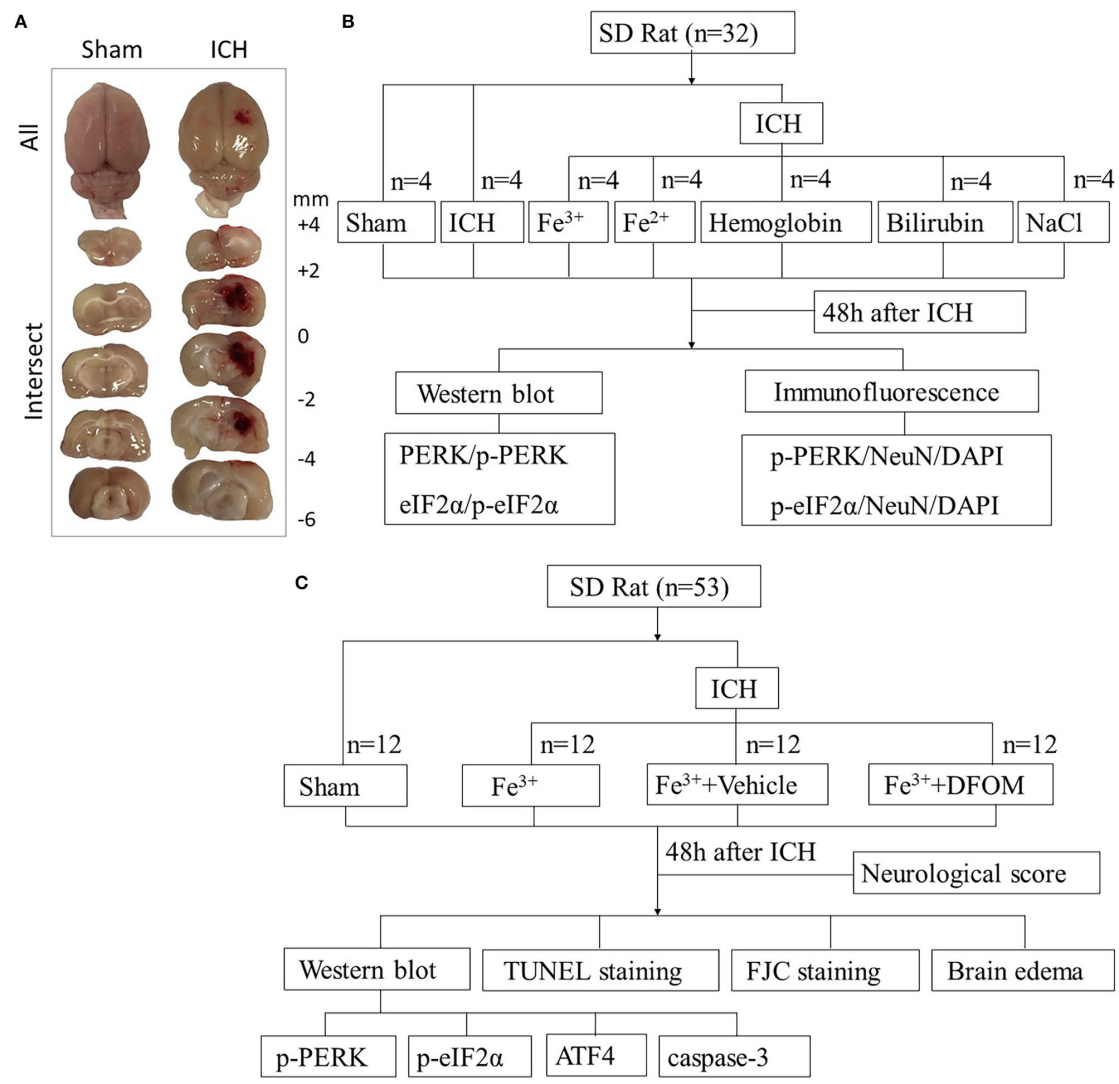
Experimental design. Brain tissues peripheral to the damaged areas in the ICH group and from the corresponding location in the Sham group were assessed **(A)**. Experiment I was to determine PERK/p-PERK, eIF2α/p-eIF2α amounts and locations following ICH, and to determine which blood components had the most impact **(B)**. Experiment II examined the effects of DFX on brain injury following ICH and explore its possible potential mechanism **(C)**.

In Experiment 1, animal weights, food intake, and motor abilities were similar in all rats. Twenty eight rats (28 out of 33) were randomized to seven groups using computer-based randomization, including Sham, ICH, ICH + Fe^3+^, ICH + Fe^2+^, ICH + Hemoglobin, ICH + Bilirubin and ICH + NaCl. NaCl was used as vehicle. At 48 h after ICH (18), the rats were sacrificed and cerebral tissues were collected around the damaged areas. Brain tissues surrounding the damaged (Figure 1A) were sampled to perform Western blot (WB) and Immunofluorescence (IF) analysis to assess the expression of PERK, p-PERK, eIF2α, p-eIF2α (Figure 1B).

In Experiment 2, in order to assess the function of DFX in ICH caused by iron, 48 rats (48 out of 52) were randomized to four groups using computer-based randomization, including Sham, ICH + Fe^3+^, ICH + Fe^3+^+Vehicle, and ICH + Fe^3+^+DFX. At 48 h after ICH, the rats were sacrificed after neurological scoring and cerebral tissues were collected around the damaged areas. Brain tissues were used for WB to measure the expression of PERK, p-PERK, eIF2α, p-eIF2α, ATF4 and caspase-3. Fluoro-jade C(FJC) and TUNEL staining were performed for detecting necrosis and neuronal apoptosis. Wet-dry method was used to measure the brain edema (Figure 1C).

### Animals

Here, totally 85 male Sprague-Dawley rats (250–300 g) were purchased from JOINN Laboratories, Suzhou, China. Of these, 76 were analyzed. Animals were housed in a controlled temperature and humidity room with a 12 h light/dark cycles and free access to food and water. The animal studies had approval from the Institutional Animal Care and Use Committee of Nanjing University of Chinese Medicine, and followed all relevant national and international guidelines.

### ICH Model

The ICH model in rats was established by autologous whole blood stereotaxic injection or several kinds of blood components according to previous reports with some modifications (11, 20). In brief, rats were anesthetized with intraperitoneally administered pentobarbitone (40 mg/kg) and mounted on a stereotaxic apparatus (SA-100, Shanghai Yuyan Instruments, China). A cranial burr hole (0.2 mm anterior to bregma, 3.5 mm lateral to midline and 5.5 mm ventral to cortex) was drilled corresponding to the right basal ganglia. After collected by cardiac puncture, autologous whole blood (100 μL) was injected slowly (20 μL/min) into basal ganglia with a microinjector (Hamilton, USA). At the same time, FeCl_3_ (1 mmol/L, 100 μL), FeCl_2_ (1 mmol/L, 100 μL), Hemoglobin (300 mg/mL, 100 μL), Bilirubin (12 mg/mL, 100 μL) and NaCl (100 μL) were also injected under the same conditions (11). Kept the needle in place for 5 min. After sealing the burr hole with bone wax, the scalp was disinfected and sutured. Rats in Sham group were only subjected to a cranial burr hole without injection. The rats were sacrificed 48 h after ICH according to previous study (18).

### Drug Injection

DFX (iron chelator, 100 mg/kg, MCE, USA) was intraperitoneally injected 2 and 6 h after ICH, followed by every 12 h until the rats were sacrificed for the material. DFX can bind free iron in a stable compound, preventing it from taking part in chemical reactions. The Fe^3+^+vehicle group was given NaCl for the same time course and dosage.

### Western Blot

WB was performed as described (21). Briefly, cortex tissues were lysed with RIPA Lysis Buffer containing Protease inhibitor cocktail (Beyotime, China), and centrifuged at 12,000 g at 4°C for 15 min. The supernatant was collected and determined with Pierce^TM^ BCA Protein Assay Kit (Thermo Fisher, USA). After mixed with 5X loading buffer (Beyotime, China), the supernatant was bathed at 100°C for 5 min. Equal amounts of extracted proteins were separated by SDS-polyacrylamide gels (Beyotime, China) and transferred onto PVDF membranes (Millipore, USA). After blocking with QuickBlock™ Blocking Buffer (Beyotime, China) at room temperature (RT) for 30 min, membranes were incubated at 4°C overnight with the following rabbit primary antibodies: anti-PERK (Abcam, USA), anti-p-PERK (Cell Signaling, USA), rabbit anti-eIF2α (Abcam), anti-p-eIF2α (1:500, Abcam), anti-ATF4 (1:1,000, Abcam) and anti-caspase-3 (1:500, Abcam). Mouse anti-β-actin (1:10,000, Sigma, USA) was used as control. Then the membranes were incubated with goat anti-rabbit IgG-HRP (Invitrogen, USA) and goat anti-mouse IgG-HRP (Invitrogen) for 1 h at RT. Detection used Immobilon^TM^ Western Chemiluminescent HRP Substrate (Millipore) and a LUMINESCENT IMAGE ANALYZER (GE Healthcare Bio-Sciences, Sweden). ImageJ (National Institutes of Health, USA) was used to analyze data.

### Immunofluorescence

Double IF staining was performed as described (22). Briefly, brain tissues were fixed with 4% paraformaldehyde, embedded in paraffin, and cut into 5 μm. After dewaxing, the sections were permeabilized in Immunostaining Permeable Buffer (Beyotime). After three washes in PBS, the sections were blocked in Immunostaining Blocking Buffer (Beyotime) for 1 h at RT and incubated with primary antibodies at 4°C overnight: rabbit anti-p-PERK (Abcam), rabbit anti-p-eIF2α (Abcam), mouse anti-NeuN (Abcam). After three washes in PBS, the sections were incubated with donkey anti-rabbit IgG Alexa Fluor 488 (Invitrogen) and donkey anti-mouse IgG Alexa Fluor 555 (Invitrogen), for 1 h at RT. Finally, counterstaining was carried out with DAPI and a U-RFL-T fluorescence microscope (OLYMPUS, Japan) was utilized for analysis.

### TUNEL Staining

TUNEL staining was carried out to detect apoptosis in intracerebral cells (18). The sections were dewaxed and incubated in proteinase K at 37°C for 20 min. After three washes in PBS, the sections were incubated in TUNEL detection liquid (Beyotime) at 37°C for 1 h. After counterstained with DAPI, the TUNEL-positive neurons were observed under a U-RFL-T fluorescence microscope (OLYMPUS).

### Fluoro-Jade C Staining

FJC staining was performed according to the manufacturer's instructions (Biosensis, South Australia). Briefly, the sections were dewaxed and incubated with potassium permanganate (1:10 in distilled water) for 10 min. Rinse the sections for 2 min in distilled water. Then the sections were incubated with FJC (1:10 in distilled water) in the dark for 10 min. After 3 rinses for 1 min in distilled water, the sections were dried at 60°C for 5 min, soaked in xylene for 1 min, and coverslipped with Neutral Balsam Mounting Medium (Yeasen Biotech. China). The FJC-positive cells were photographed by a U-RFL-T fluorescence microscope (OLYMPUS) and the total number of FJC-positive cells was the average of three high-power fields.

### Brain Edema

The wet-dry method was carried out for assessing brain edema in the injured brain (23, 24). Briefly, the brains were separated into symmetrical sides and their wet weights were immediately obtained. Then the samples underwent drying at 100°C for 72 h (or until the dried weights no longer change), followed by dry weight measurements. Brain water content (%) was derived as [(wet weight-dry weight)/(wet weight)] × 100%.

### Neurological Scoring

Neurological deficiency was conducted at 48 h post-ICH based on the modified Garcia score consisted of seven components (25, 26). Scores in each subtest ranged between 0 and 3 (maximum total score of 21, indicating no neurological defects).

### Statistical Analysis

All data are mean ± SD. Data analysis was carried out with GraphPad Prism 8.0 software (San Diego, USA). One-way ANOVA was used for multiple comparisons, and Student–Newman–Keuls *post-hoc* test was used to determine the differences between groups. *p* < 0.05 indicated statistical significance.

## Results

### PERK/p-PERK, eIF2α/p-eIF2α Protein Levels in Brain Tissues Following ICH Caused by Several Blood Components

**Figure 2:**
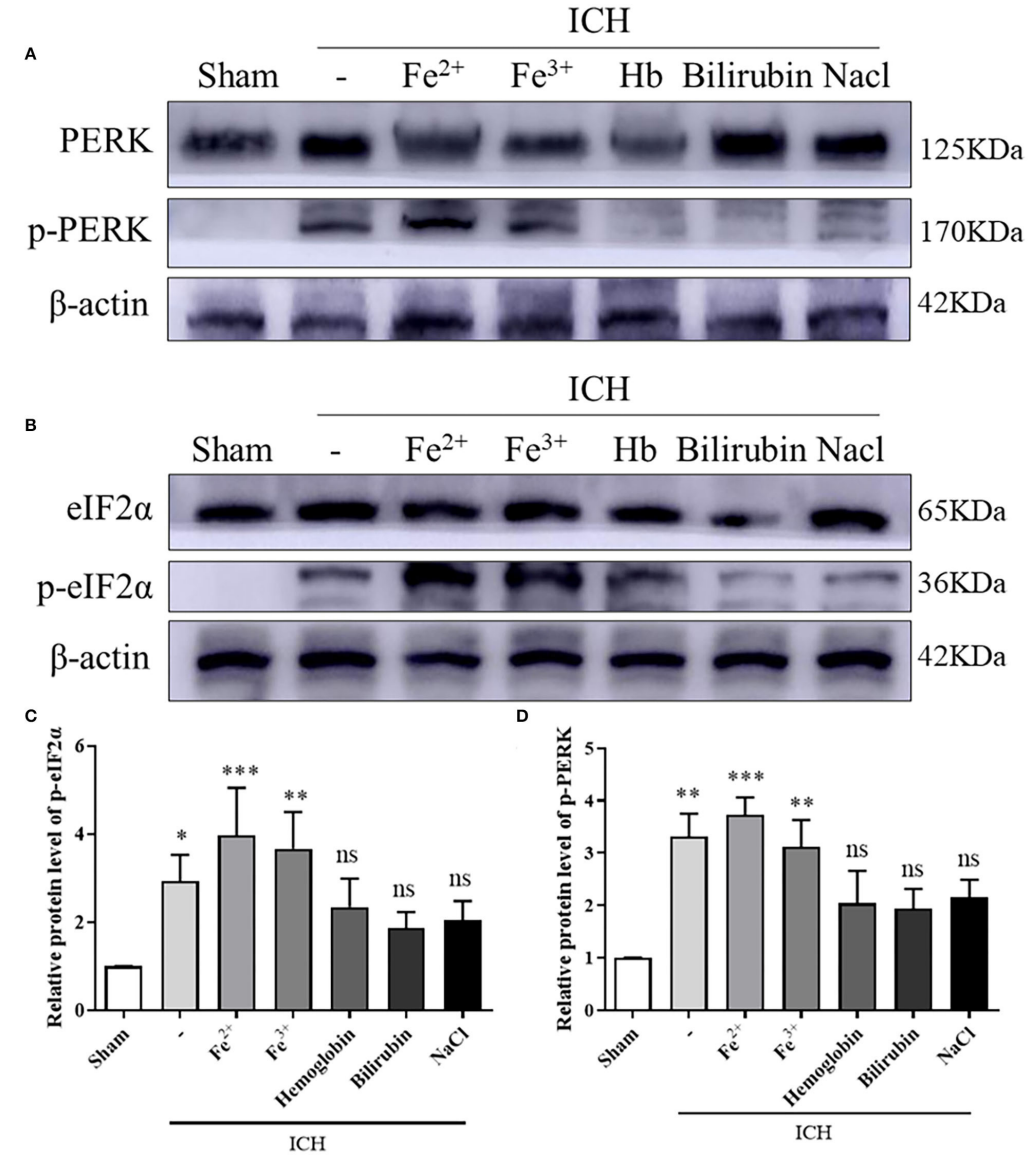
PERK/p-PERK and eIF2α/p-eIF2α protein amounts in peri-injury cortex following ICH. WB analysis and quantification of p-PERK **(A,C)** and p-eIF2α **(B,D)** protein amounts in ICH caused by whole blood, FeCl_3_, FeCl_2_, hemoglobin(Hb), bilirubin, and saline in brain tissues. The Sham group was used for normalization, and quantitation used ImageJ. Data was mean ± SD (*n* = 4). *p < 0.05, **p < 0.01, ***p < 0.005, ^ns^*p* > 0.05 vs. Sham.

**Figure 3:**
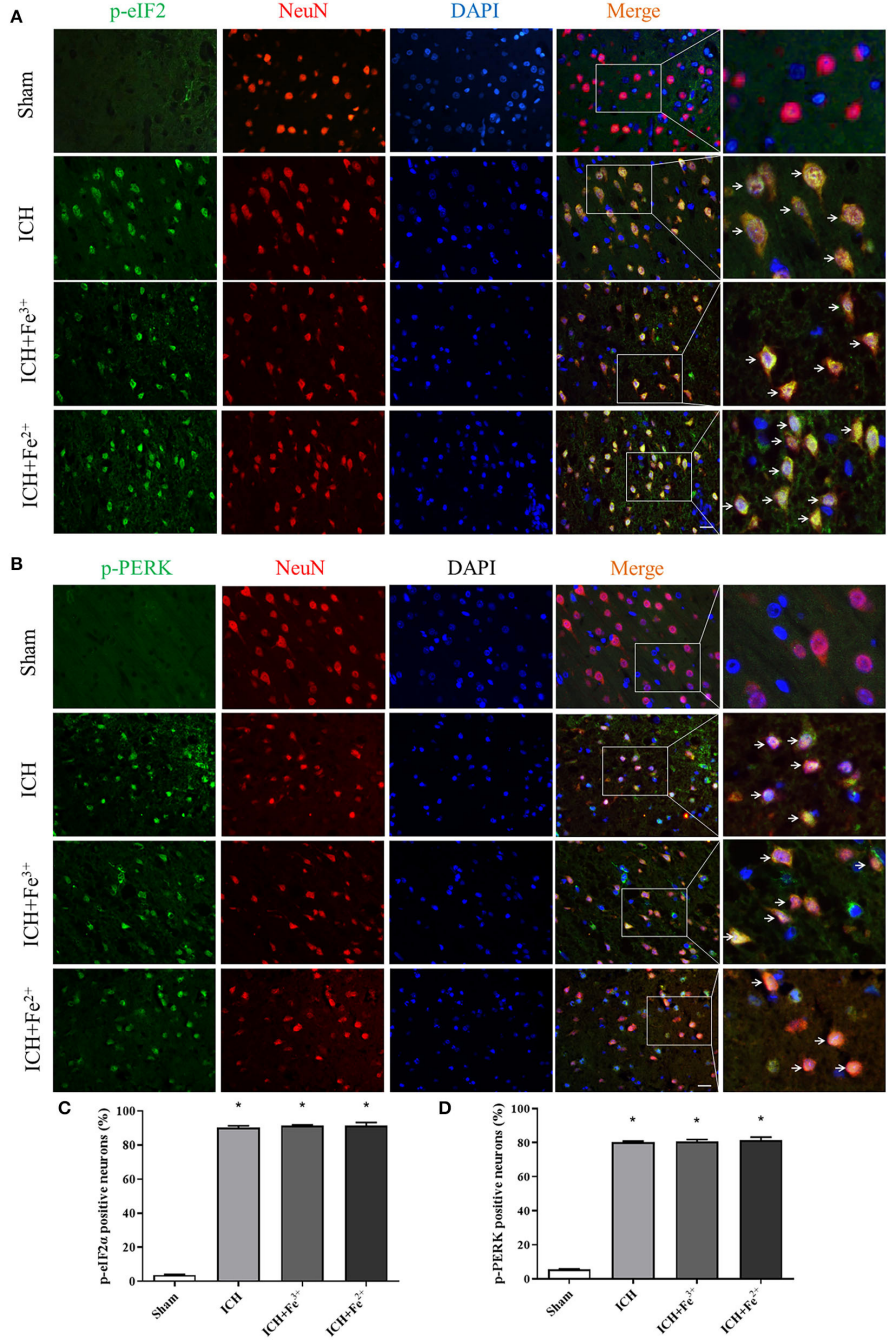
Post-ICH IF in rat brain tissues. Double immunofluorescence micrographs showing green-labeled p-eIF2α **(A,C)**/p-PERK **(B,D)** and red-labeled NeuN neurons surrounding the damaged brain tissue. Counterstaining utilized DAPI (blue). Scale bar = 50 μm. Data was mean ± SD (*n* = 4). *p < 0.05 vs. Sham.

WB showed that, compared with the Sham group, p-PERK and p-eIF2α protein amounts increased in the ICH, ICH + Fe^3+^, and ICH + Fe^2+^ groups. However, other groups demonstrated little changes (Figure 2). Consistent with WB analysis, IF staining revealed that there were numerous p-PERK-positive neurons and p-eIF2α-positive neurons in ICH, ICH + Fe^3+^, and ICH + Fe^2+^ groups at 48 h following ICH compared with the Sham group (Figure 3).

### DFX Intervention Through the Perk Pathway Following ICH

**Figure 4:**
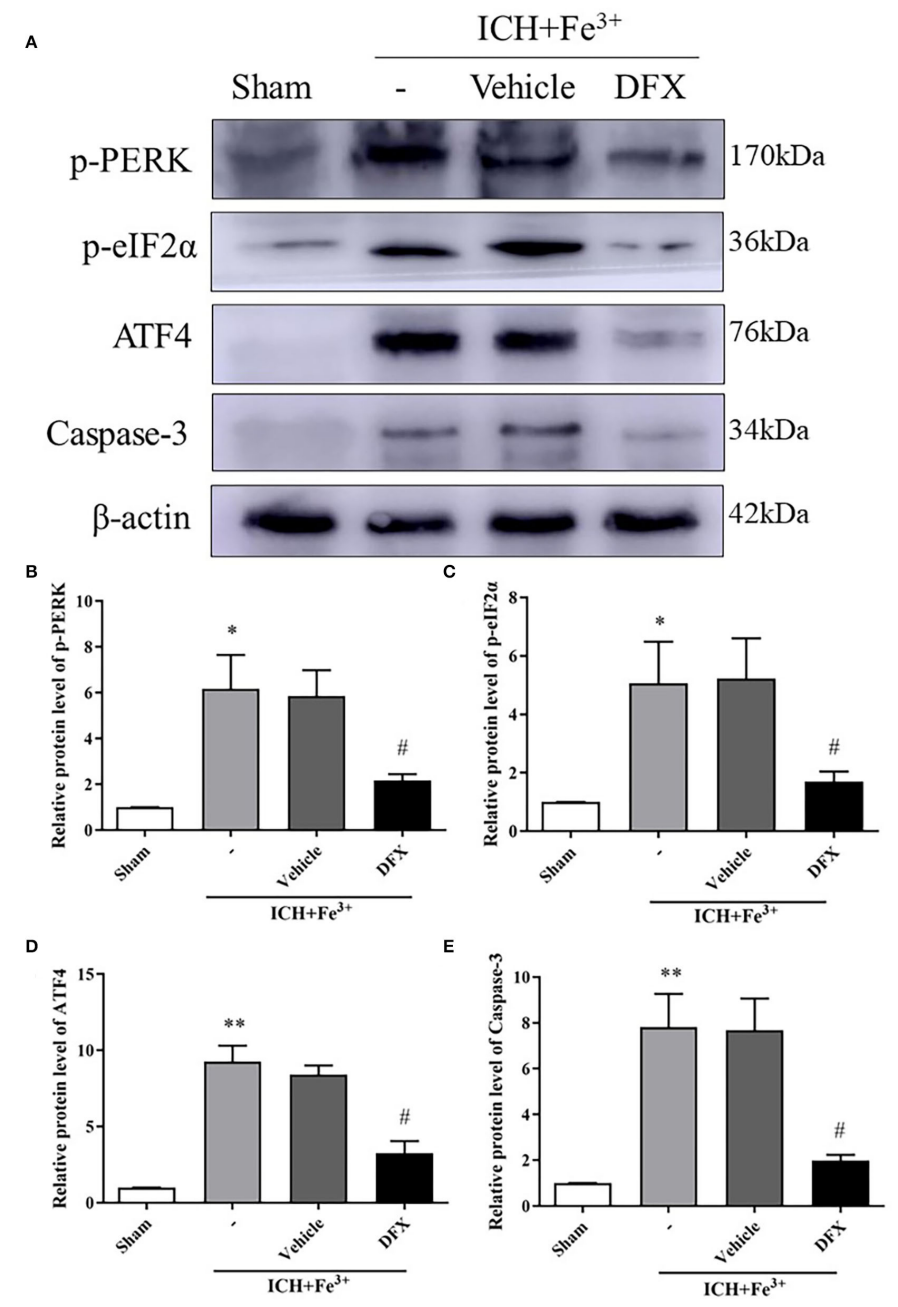
Effect of the intervention of DFX following ICH. WB analysis and quantification of p-PERK **(A,B)**, p-eIF2α **(A,C)**, ATF4 **(A,D)** and Caspase-3 **(A,E)** protein levels in ICH caused by Fe^3+^ in brain tissues. The Sham group was used for normalization, and quantitation used ImageJ. Data was mean ± SD (*n* = 4). *p < 0.05, **p < 0.01 vs. Sham, #*p* < 0.05 vs. ICH+Fe^3+^ group.

After DFX intervention, the protein level of p-PERK and p-eIF2α was significantly decreased in the Fe^3+^+DFX group compared with the ICH + Fe^3+^ group. The protein level of Fe^3+^+Vehicle group was similar to the ICH + Fe^3+^ group. Additionally, ATF4 protein levels were higher in the Fe^3+^ and Fe^3+^+Vehicle groups than in the Sham group. After DFX intervention, ATF4 level was significantly lower in the Fe^3+^+DFX group compared with the ICH + Fe^3+^ group (Figure 4).

### Neuronal Degeneration and Neuronal Death Following ICH in Rats Administered DFX

**Figure 5:**
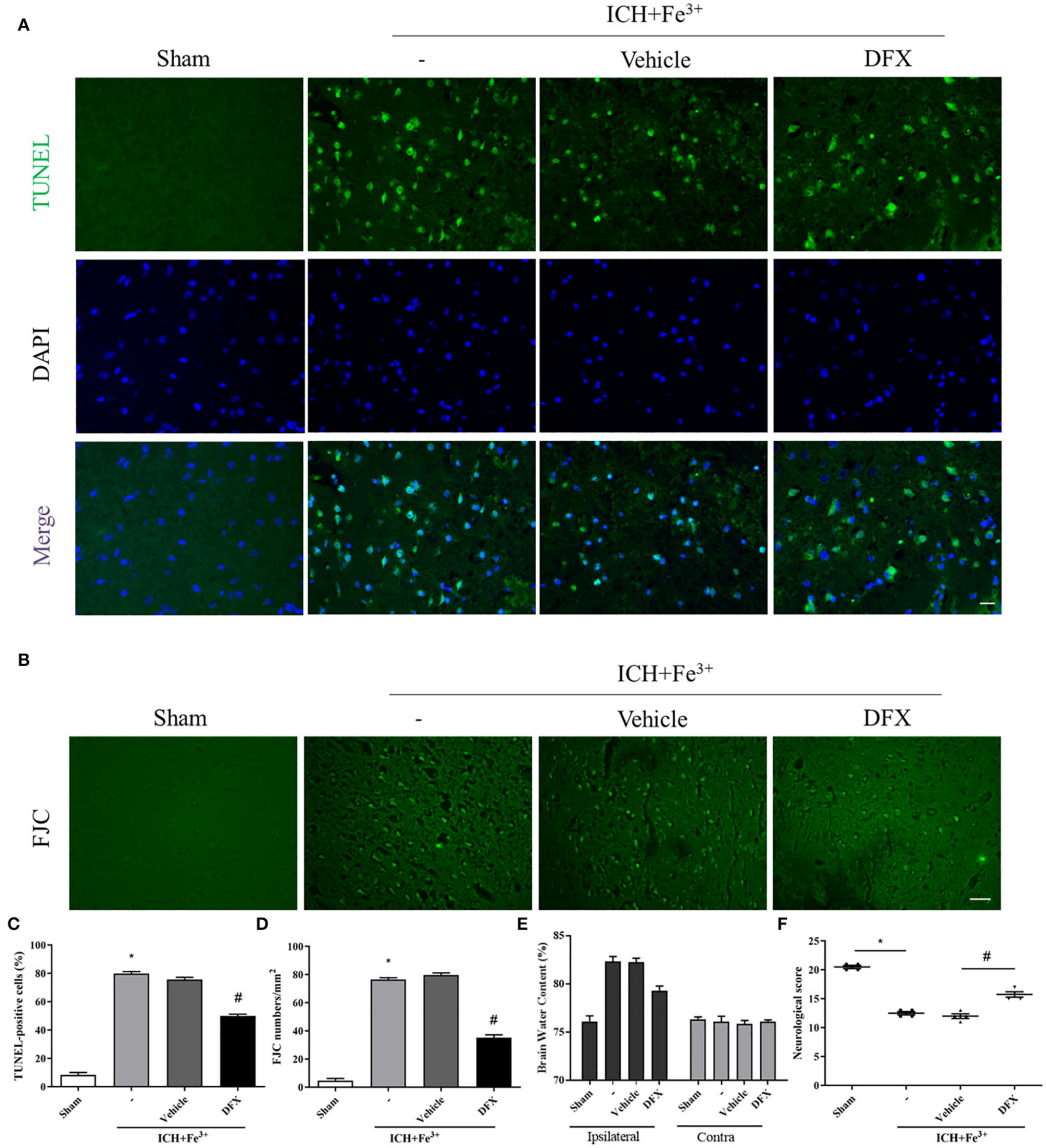
Effect of the intervention of DFX on PERK signaling pathway 48 h following ICH. TUNEL-positive neurons **(A,C)** in each group following DFX administration. Scale bar = 50 μm. FJC-positive cells **(B,D)** at 48 h in each group. Scale bar = 100 μm. Brain water amounts of the bilateral hemispheres were assessed by the wet-dry technique **(E)**. Neurological behavioral scores in each group **(F)**. Data was mean ± SD (*n* = 4). *p < 0.05 vs. Sham, #*p* < 0.05 vs. ICH+Fe^3+^ group.

Protein levels of Caspase-3 were lower following DFX intervention when compared to the ICH + Fe^3+^ group (Figures 4A,E). Additionally, the degree of neuronal apoptosis (TUNEL) in the ICH + Fe^3+^ group was significantly greater in comparison with that of Sham group. There was no difference in neuronal apoptosis between the ICH + Fe^3+^ and Fe^3+^+Vehicle groups, while it was significantly lower in the Fe^3+^+DFX group when compared to the ICH + Fe^3+^ group (Figure 5A). The degree of neuronal necrosis (FJC) was consistent with the degree of neuronal apoptosis (Figure 5B).

### Neurological Behavioral Scores and Brain Edema in ICH Rats Following DFX Intervention

Neurological behavioral scores from a modified Garcia test were starkly decreased in the ICH + Fe^3+^ group when compared to the Sham group, while the ICH + Fe^3+^ group and Fe^3+^+Vehicle group had comparable scores. The Fe^3+^+DFX group had overtly ameliorated neurological behavioral scores in comparison with the ICH + Fe^3+^ group (Figure 5F). Additionally, brain edema was markedly reduced in the injured hemispheres following DFX intervention post-ICH. However, brain edema had no significant changes in the contralateral brain (Figure 5E).

## Discussion

**Figure 6:**
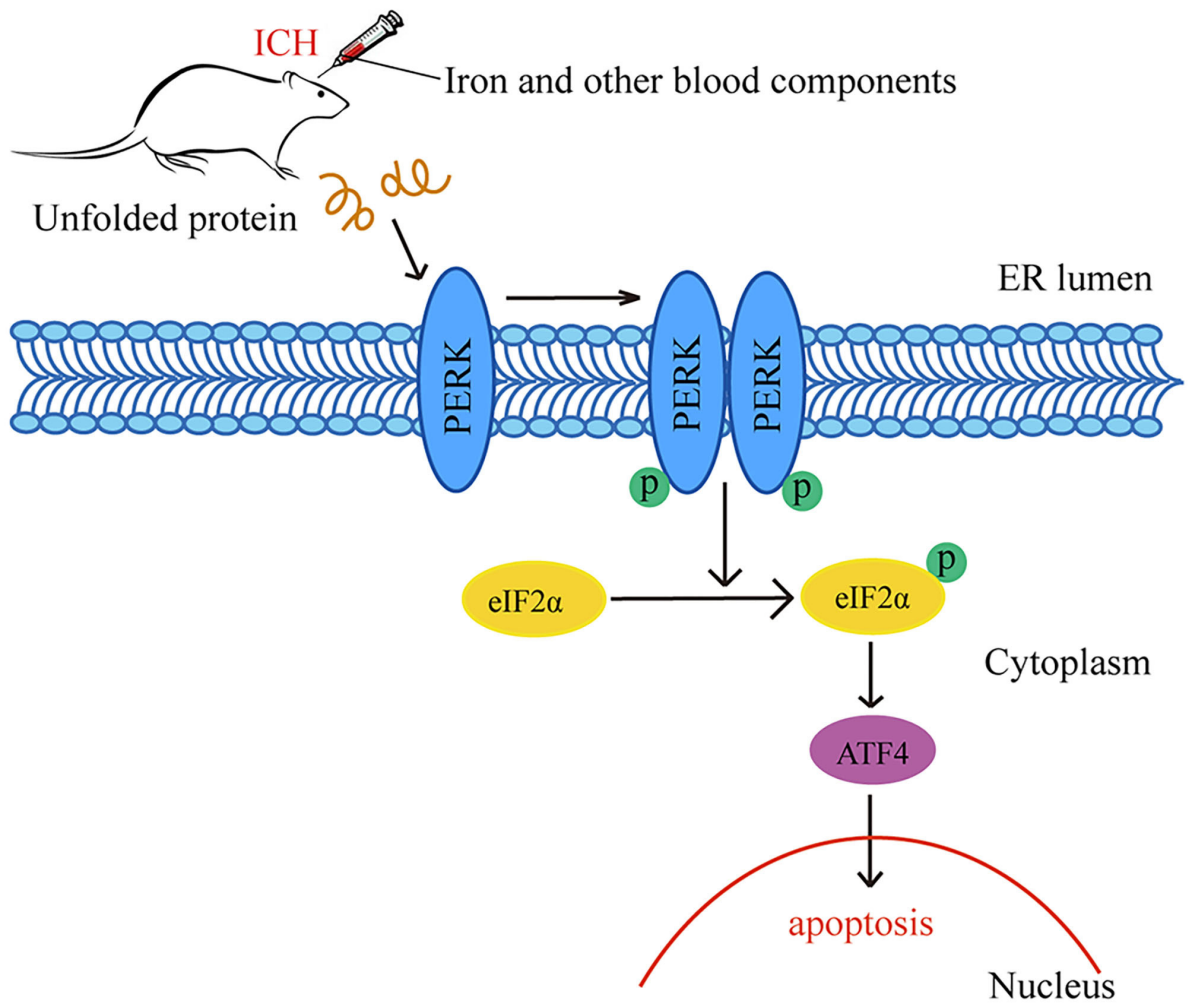
Schematic representation of potential mechanisms of the neuroprotective effects of PERK pathway after ICH. Following ICH caused by autologous whole blood or other blood components (especially Fe^3+^ and Fe^2+^), the activation of ER stress activates the PERK pathway and increases the protein amounts of p-PERK, p-eIF2α and ATF4. The activation of PERK pathway blocks the initiation process of translation and synthesis of protein, leading to neuronal apoptosis.

In this study, we found that iron plays a significant role in the occurrence of ICH-induced SBI. After ICH caused by autologous whole blood or other blood components (especially Fe^3+^ and Fe^2+^), ERs activated the PERK pathway and increased p-PERK, p-eIF2α and ATF4 protein levels. The PERK pathway blocked the initiation process of translation and synthesis of protein, leading to neuronal apoptosis (Figure 6). We used DFX to interfere with Fe^3+^. It showed that DFX could prevent neuronal apoptosis by inhibiting the PERK pathway and alleviate ERs.

Studies have reported that, ERs-induced neuronal death is one of the important causes of brain injury. Cells activate a series of adaptive processes through UPR to restore the function of ER when ERs occurs. As a part of the UPR, the PERK pathway may be associated with a series of pathophysiological processes in neurological diseases (27). As we have previously reported, in early ICH-induced SBI, PERK activation is related to oligomerization and trans-autophosphorylation when unfolded or misfolded proteins accumulate, and p-PERK specifically induces eIF2α to p-eIF2α at ser51 to upregulate ATF4 (18, 19). However, it is unclear which components of blood are involved in the development of ICH through the PERK pathway. Previous studies did not address the effects of various blood components on post-ICH neuronal apoptosis via the PERK pathway. Therefore, in this study, we explored these effects. Specifically, we investigated the pathophysiological role of multiple components produced after hematoma decomposition in ICH and the relationship between blood components and the PERK pathway.

As we all know, blood components include hemolyzed erythrocytes, methemoglobin, ferritin, chloride, fibrinogen, thrombin, and hemin (28). The effects of hemoglobin, iron, and other blood components on brain tissue in damaged areas are considered to be the influencing factors of SBI. Brain edema was observed after the injection of lysed blood cells into the ICH model, while not observed following injection of packed erythrocytes, suggesting the lysed products of RBC were the main cause of brain injury (29). In our study, we used hemoglobin, iron (Fe^3+^ and Fe^2+^) and bilirubin to conduct an ICH rat model. Protein expressions of p-PERK and p-eIF2α were increased in the iron-treated rats. Which was consistent with it in the rats treated with autologous whole blood (Figure 2). This suggests that iron plays a role after ICH, and this is consistent with previous reports.

Here, we found that both Fe^2+^ and Fe^3+^ can cause brain injury. The p-PERK, p-eIF2α and ATF4 expression can be reduced by the use of DFX chelating iron to silence the PERK pathway, and reduce the brain injury (Figure 4). It has been reported that, blood components such as iron and thrombin play a significant role in brain injury following ICH (30). RBC lysate contains hemoglobin, as well as its components, iron and heme. The form of the iron within heme can be either Fe^2+^ or Fe^3+^, both of which can cause brain injury (31). Under normal conditions, iron is present in the form of Fe^3+^ under dynamic regulation by iron transporters on cell membranes, or stored on proteins to form transferrin and ferritin. However, in the case of pathological damage to the regulatory system of the cell, the increased Fe^3+^ is readily converted to Fe^2+^ by the electron transfer response (32). The degradation of hemoglobin causes the release of iron when RBCs are lysed. The concentration of iron surrounding the perihematomal brain tissue can reach as high as 10 mmol/L, causing obvious brain damage (33). Based on previous studies, we demonstrated that iron can cause more severe nerve damage and brain edema compared to a variety of other blood components (Figure 5). Therefore, we selected iron as the intervention target, and combined DFX with Fe^3+^ to explore the effect of these on brain injury and brain edema through the PERK pathway. This points out the importance of Fe^3+^ in hydrocephalus and brain damage after erythrolysis.

Neuronal necrosis, apoptosis, and destruction of the BBB are the main components of brain injury following ICH. The destruction of the BBB can lead to the formation of brain edema, neuronal death, and neurological impairment. Iron from blood degradation is associated with BBB dysfunction after hemorrhagic stroke (34). It has shown that intraventricular injection of iron can cause hydrocephalus. The chelation of iron and DFX is able to reduce hydrocephalus caused by RBC lysis (11). In this study, following ICH with the injection of iron, BBB permeability was increased, and brain edema was aggravated, leading to neuronal necrosis and apoptosis. Using DFX following ICH can reverse these results, resulting in reduced cerebral edema, nerve necrosis, and apoptosis (Figure 5).

The current study has several limitations. First, in this study, we only focused on adult male rats, despite ICH being common in older females (35). Second, we focused on the PERK pathway, ignoring the other two UPR pathways (IRE1 pathways and ATF6 pathway). Third, iron comes in two forms, Fe^3+^ and Fe^2+^, and both are important during ICH. However, we only used DFX to chelate Fe^3+^, as Fe^2+^ does not have a specific inhibitor. In future research, we can attempt to find more specific methods to intervene Fe^2+^ to further explore its effect on ICH. Additionally, the specific initiation mechanism of the UPR pathway following ICH also needs further study.

In conclusion, this study provides evidence that various blood components, especially iron, the lysed product of RBCs, can lead to neuronal apoptosis and induce brain edema. This may be an effective therapeutic target for ICH. Furthermore, the effects of iron in SBI following ICH may be related to the PERK pathway.

## Data Availability Statement

The original contributions generated for this study are included in the article/supplementary material, further inquiries can be directed to the corresponding author/s.

## Ethics Statement

The animal study was reviewed and approved by Institute of Animal Care Committee of Zhangjiagang Traditional Chinese Medicine Hospital.

## Author Contributions

BD and GC: study conception and design. MW and RG: data collection and analysis. MW: writing of the manuscript. All authors have assessed the totality of study data, and are responsible for their integrity and accurate analysis.

## Conflict of Interest

The authors declare that the research was conducted in the absence of any commercial or financial relationships that could be construed as a potential conflict of interest.
